# The Role of Androgen Supplementation in Women With Diminished Ovarian Reserve: Time to Randomize, Not Meta-Analyze

**DOI:** 10.3389/fendo.2021.653857

**Published:** 2021-05-17

**Authors:** Ana Raquel Neves, Pedro Montoya-Botero, Nikolaos P. Polyzos

**Affiliations:** ^1^ Department of Obstetrics, Gynecology and Reproductive Medicine, Dexeus University Hospital, Barcelona, Spain; ^2^ Faculty of Medicine, Autonomous University of Barcelona, Cerdanyola del Vallès, Spain; ^3^ Department of Reproductive Medicine, Conceptum – Unidad de Fertilidad del Country, Bogotá, Colombia; ^4^ Faculty of Medicine and Health Sciences, Ghent University (UZ Gent), Gent, Belgium

**Keywords:** androgens, testosterone, DHEA, poor ovarian response (POR), diminished ovarian response (DOR)

## Abstract

The management of patients with diminished ovarian reserve (DOR) remains one of the most challenging tasks in IVF clinical practice. Despite the promising results obtained from animal studies regarding the importance of androgens on folliculogenesis, the evidence obtained from clinical studies remains inconclusive. This is mainly due to the lack of an evidence-based methodology applied in the available trials and to the heterogeneity in the inclusion criteria and IVF treatment protocols. In this review, we analyze the available evidence obtained from animal studies and highlight the pitfalls from the clinical studies that prevent us from closing the chapter of this line of research.

## Introduction

In women, testosterone and dihydrotestosterone (DHT), the bioactive androgens that bind directly to the androgen receptor (AR), are produced by peripheral conversion of androgen precursors (androstenedione, dehydroepiandrosterone and dehydroepiandrosterone sulfate) that are secreted from both the ovary and adrenal gland (1, 2).

The AR is expressed at all levels of the female hypothalamic-pituitary-gonadal axis (2). In the ovary, the AR has been detected in several stages of oocyte development from the primary stage onwards, as well as in the ovarian stroma (3). The fact that hyperandrogenic women present an increased number of small antral follicles suggests a role for androgens in both follicular development and follicular arrest. Clinical examples of this effect include polycystic ovarian syndrome (PCOS) and congenital adrenal hyperplasia patients (4). On the other hand, although initial studies using histomorphologic criteria suggested that exposure to exogenous testosterone treatment in female-to-male transexual patients induced polycystic ovary morphology (5, 6), more recent studies using both histologic and ultrasound criteria have not confirmed these findings (7–9).

Circulating androgen levels have been reported to decline with age, especially during the earlier reproductive years (10). Similarly, the reproductive aging process consists of a gradual reduction in oocyte quantity and quality, with a consequent age-related decrease in the reproductive potential (11, 12). In the light of these findings, IVF centers have initiated androgen pretreatment in patients with diminished ovarian reserve, intending to improve their reproductive outcomes. In fact, a recent survey has shown that more than 40% of physicians in Europe and Australia are prescribing off-label androgens in this subgroup of patients (13). However, the evidence for including this approach in our clinical practice is scarce.

The aim of this review is to analyze the available evidence from animal studies regarding the impact of androgen supplementation on folliculogenesis, as well as the drawbacks from clinical studies that might preclude the obtention of definitive conclusions to guide an evidence-based approach for such a challenging population.

## Methods

The Cochrane Central Register of Controlled Trials (CENTRAL), MEDLINE *via* PubMed, the Web of Science and Scopus were screened with a combination of keywords related to ART, poor responders, diminished ovarian response, androgens, testosterone and DHEA in various combinations. The search period was from the date of inception of each database until 1 December 2020. Only full text papers published in English were included.

## The Promising Evidence From Animal Studies

### Primordial Follicle Initiation

Previous studies in primates have shown that androgens ﻿increase the numbers of small- and medium-sized follicles but not large preovulatory follicles (14). In particular, testosterone and DHT pretreatment increased the number of primary follicles. Also, they resulted in a significant increase in insulin growth factor I (IGF-I) and IGF-I receptor mRNAs in the oocytes of primordial follicles, suggesting that androgen-induced activation of oocyte IGF-I signaling may trigger primordial follicle growth (15). More recently, mouse studies have corroborated that testosterone promotes primordial follicle to primary follicle transition *via* an AR-mediated pathway rather than by transformation into estradiol (16).

### Preantral to Antral Stage Transition

Besides the effect on primordial follicle initiation, androgens also seem to have a role in the preantral to antral stage transition. In vivo studies in ovine models have shown that ﻿DHEA exposure stimulates early follicular growth during the preantral and early antral follicular stages (17). Studies in mouse models have also shown that both DHT and testosterone ﻿stimulate granulosa cell (GC) proliferation and both secondary and preantral follicle growth (18). Moreover, ﻿androgens seem to support follicle development during the FSH-dependent preantral stage by increasing the expression of FSH receptor mRNA levels and, therefore, enhancing FSH action (19, 20). ﻿GC-specific AR-null mice experiments have also shown that ﻿AR signaling in GCs is necessary for progression beyond the preantral stage (21). Androgens enhance antiapoptotic pathways, thereby contributing to follicle survival, and ﻿improve sensitivity to FSH-induced follicle growth and progression to the antral stage (22). On the other hand, when AR signaling is blocked, preantral follicles cannot progress to antral follicles and, instead, are subjected to an increased rate of atresia.

### The Peri-Ovulatory Stage

The effect of androgens in later stages of follicle development, namely in the pre- and peri-ovulatory stage, is controversial. Studies in primates have shown that testosterone treatment did not increase the number of preovulatory follicles (14). However, experiments in pigs have shown that androgens might have regulatory functions during late follicular development (23). In fact, DHT treatment resulted in an increase in the amount of FSH receptor mRNA in preovulatory follicles and increased ovulation rate (23). Similarly, experiments in mice have also shown that ﻿testosterone has a role in the maturation of oocytes arrested in prophase I of meiosis (24) and that DHT significantly increased the number of ovulated oocytes (22). On the other hand, Romero and Smitz reported that elevated levels of androstenedione and testosterone negatively affected meiotic resumption (25). These conflicting findings regarding the role of androgens in the late stages of follicular development suggest that further studies are needed to clarify the physiopathology behind such complex interactions.

**Figure 1:**
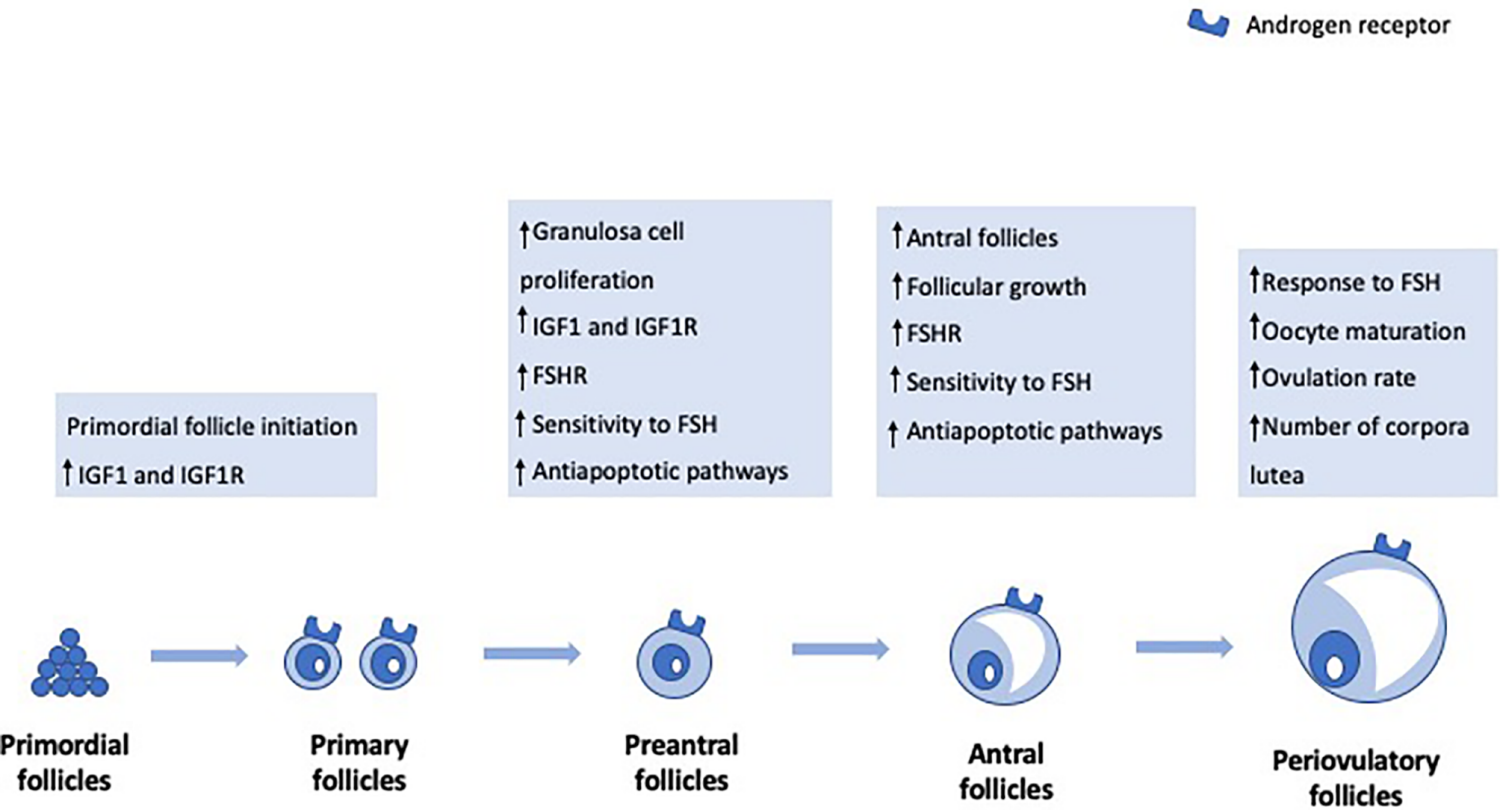
Androgen effects on folliculogenesis.

**Figure 1** highlights the main androgen effects on folliculogenesis.

### Genetic Studies

Finally, data from genetic models have also reaffirmed the role of AR-mediated activity in the regulation of ovarian function. Studies using ﻿female mouse models homozygous for an inactivated AR (ARKO) have revealed reduced fertility and a defective folliculogenesis (26–28), as well as a reduced litter size (27), increased follicular atresia and premature ovarian failure (21). Together, these data suggest the AR signaling pathway mediates both intra and extra-ovarian actions, with an essential role in maintaining normal ovarian function and fertility.

## The Pitfalls From Clinical Studies

All these promising data obtained from animal studies and the fact that both androgens and ovarian reserve decline steeply with age, led to the speculation that androgen replacement in women with DOR might delay these age-related effects. ﻿However, despite several lines of evidence supporting a role for androgens in folliculogenesis, the available data from clinical studies remains unconvincing. This might be related to the methodological inconsistencies observed in the available trials (**Tables 1** and **2**).

**Table 1:** Published randomized controlled trials on the use of DHEA and Testosterone in DOR and POR patients.

Author *Year*	Definition of POR	Number of patients	Dose	Duration	Stimulation protocol	Primary outcome
**Testosterone**						
Massin et al. (29) *2006* *	Previous POR (Peak E2<1200pg/mL and ≤5 oocytes) and D3 FSH > 12 IU/L or E2 > 70pg/mL or Inhibin B <45ng/mL	49	10 mg/d	15-20 d	﻿NR	Total number of retrieved oocytes
Fábregues et al. (30) *2009*	Previous POR and 31-39y	62	20 ug/kg/d	5 d	Long GnRH agonist	﻿Incidence of low responders
Kim et al. (31) *2011*	Previous cycle with ≤3 oocytes retrieved despite high Gn dose	110	12.5 mg/d	21 d	GnRH antagonist	Number of MII oocytes retrieved
Kim et al. (32) *2014*	Previous cycle with ≤3 oocytes retrieved despite high Gn dose	120	12.5 mg/d	I1: 14 d/ I2: 21 d/ I3: 28 d	GnRH antagonist	Number of MII oocytes retrieved
Marzal Escrivá et al. (33) *2015*	≥2: ≥38y, AFC ≤6, FSH ≥10 IU/L, AMH ≤5pg/mL AND ﻿≤4 follicles of ≥16 mm on the day of trigger or ﻿E2 ≤500 pg/mL on the day of trigger or ≤ 4 MII	66	20 ug/kg/d	7 d	GnRH antagonist	Number of MII oocytes retrieved
Bosdou et al. (34) *2016*	Bologna criteria	50	10 mg/d	21 d	Long GnRH agonist	Total number of retrieved oocytes
Saharkhiz et al. (35) *2018* *	Bologna criteria	48	25 mg/d	During COS	GnRH antagonist	NR
**DHEA**						
Wiser et al. (36) *2010*	<5 oocytes retrieved in previous cycle; poor quality embryos; previous cycle cancelation due to poor response with rFSH 300IU	33	75 mg/d	> 6 weeks	Long GnRH agonist	Peak estradiol levels, the number of retrieved oocytes, embryo quality and number of embryos reserved for transfer
Artini et al. (37) *2012*	Bologna criteria	24	75 mg/d	12 weeks	GnRH antagonist	HIF1 and VEGF concentrations in the FF and the number of MII oocytes
Moawad and Shaeer (38) *2012*	<40y; <5 oocytes retrieved in previous cycle; previous cycle cancelation due to poor response with rFSH 300IU; AMH<1.7ng/mL	133	75 mg/d	>12 weeks	GnRH antagonist	Peak E2 levels, number of retrieved oocytes and number of embryos
Yeung et al. (39) *2013* *	POI	22	75 mg/d	16 weeks	NA	Serum AMH level
Yeung et al. (40) *2014* *	<40y, subfertility >1y and AFC<5	32	75 mg/d	12 weeks	GnRH antagonist	The primary outcome was the AFC at 12 weeks
Kara et al. (41) *2014*	AMH<1ng/mL or FSH>15IU/L and AFC < 4	208	75 mg/d	12 weeks	Microdose flare	NR
Zhang et al. (42) *2014*	D3 FSH ≥ 10IU/L or FSH/LH>3; AFC<5; previous cycle with <5 oocytes retrieved or previous cancelled cycle due to POR	95	75 mg/d	12 weeks	HMG + Clomiphene citrate	Follicular fluid BMP- 15 and GDF-9 and serum AMH, FSH and E2
Kotb et al. (43) *2016*	Bologna criteria 25-40y	140	75 mg/d	3 months	GnRH antagonist	Clinical pregnancy rate
Agarwal et al. (44) *2017* *	18-45y with DOR: (1) FSH levels >7 mIU/ml for age<33y; >7.9 mIU/ml for age 33–37y; >8.4 mIU/ml for age >38 years. (2) AMH < 1.05 ng/ml. (3) AFC<4	40	75 mg/d	12 weeks	NA	AMH, FSH and AFC
Narkwichean et al. (45) *2017* *	AFC<10 and/or AMH <5 pmol/L	52	75 mg/d	>12 weeks	Long GnRH agonist	Number of oocytes retrieved
Elprince et al. (46) *2020* *	﻿(1) serum AMH < 1.1 ng/mL, (2) FSH ≥ 10 mIU/L and ≤ 15 mIU/L on cycle D3, and (3) AFC ≤ 4	50	75 mg/d	2 Continuous cycles	Ovulation induction	NR

* Placebo controlled.

AFC, antral follicle count; AMH, antimullerian hormone; BMP-15, bone morphogenetic protein-15; d, day(s); E2, estradiol; FF, follicular fluid; FSH, follicle stimulating hormone; GDF-9, growth differentiation factor-9; Gn, gonadotropin; GnRH, gonadotropin releasing hormone; HIF, Hypoxia inducible factor; MII, mature oocytes; NR, not reported; NA, not applicable; POI, premature ovarian insufficiency; POR, poor ovarian responders; VEGF, vascular endothelial growth factor; y, years.

**Table 2:** Published observational trials on the use of DHEA and Testosterone in DOR and POR patients.

Author *Year*	Study design	Definition of POR	Number of patients	Dose	Duration	Stimulation protocol	Main outcome measure
**Testosterone**							
﻿Balasch et al. (47) *2006*	Prospective self-controlled	31-39y patients undergoing their third IVF attempt with 1 or 2 previous IVF cycles cancelled because of poor follicular response, with basal FSH <10IU/L	25	2.5mg/d Patch	5 d	Long GnRH agonist	NR
Mitri et al. (48) *2016*	Retrospective	﻿At least one previous failed or cancelled IVF cycle ﻿with suspected Gn resistance (serum FSH ≥20 mIU/L on D7) and ﻿absent or minimal follicular growth during the current cycle.	26	25mg/d gel	variable	Microflare GnRH agonist with interrupted FSH	NR
Doan et al. (49) *2017*	Prospective	﻿History or probability of POR: AFC<5–7 or AMH﻿≤ 1.26 ng/ml)	110	12.5mg/d gel	28 d	GnRH antagonist	NR
Fabregues et al. (50) *2019*	Retrospective	Bologna criteria	141	2.5mg/d Patch	5 d	GnRH antagonist and Long GnRH agonist	NR
**DHEA**							
Casson et al. (51) *2000*	Case series	Previous POR to vigorous Gn stimulation (peak estradiol ≤500 pg/ml, MII ≤2)	5	80mg/d	2 months	Ovulation induction	NR
Barad and Gleicher (52) *2005*	Case report	43y patient	1	75 mg/d	11 months	GnRH agonist	﻿Peak E2 concentration, oocytes retrieved, and cyropreservable embryos.
Barad and Gleicher (53) *2006*	Retrospective self-controlled	﻿Prior IVF cycle with age-appropriate COS, and < 4 oocytes retrieved, uniformly poor embryo quality and FSH >10 mIU/ml or E2 >75 pg/ml	25	75 mg/d	17.6 ± 2.13 weeks	GnRH agonist	NR
Barad et al. (54) *2007*	Retrospective	Basal FSH <12 mIU/ml, but exceeding the 95% CI of the mean value for the patient’s age group or ﻿vasal FSH ≥12 mIU/ml and/or a baseline estradiol level ≥75 pg/ml	190	75 mg/d	﻿3.8 ± 0.3 months	Microflare GnRH agonist	Clinical pregnancy rate
Mamas and Mamas (55) *2009*	Case series	POI	5	50-75 mg/d	2-6 months	NA	NR
Mamas and Mamas (56) *2009*	Case series	POI	14	50-75 mg/d	3-7 months	NA	NR
Sonmezer et al. (57) *2009*	Prospective self-controlled	﻿(i) cycle cancellation due to E2<130 pg/ml on cycle D6 or <450 pg/ml on the day of trigger, (ii) <4 retrieved oocytes despite vigorous ovarian stimulation.	19	75 mg/d	﻿90-180 d	﻿GnRH antagonist	﻿Antral follicle count, number of follicles >14 and >17 mm on the day of HCG administration, E2 on the day of HCG administration, number of retrieved oocytes, mean number of MII, number of transferred embryos and rates of fertilization, implantation, pregnancy, and clinical pregnancy.
Gleicher et al. (58) *2009*	Retrospective	Definition of POR changed over the study period	73	75 mg/d	> 2 months	NR	Miscarriage rate
Gleicher et al. (59) *2010*	Retrospective	﻿Elevated age-specific baseline FSH or abnormally low age-specific AMH	66	75 mg/d	>4 weeks	Microflare GnRH agonist	Number and percentage of aneuploid embryos
Gleicher et al. (60) *2010*	Retrospective	﻿Elevated age-specific baseline FSH or universal AMH < 0.8 ng/ml	120	75 mg/d	﻿73 ± 27 d	NA	AMH
Weissman et al. (61) *2011*	Retrospective self-controlled	﻿>1 of the following characteristics in a previous cycle with high-dose Gn stimulation:< 5 oocytes retrieved, ≤ 3 follicles ≥ 16 mm on the day of cycle cancelation, or E2 level <500 pg/ml on the day of trigger	15	75 mg/d	~3 months	NR	﻿Progesterone concentration on day 5 of stimulation and on the day of hCG administration.
Fusi et al. (62) *2013*	Prospective	Cohort 1: Previous IVF cycle with POR Cohort 2: > 40y and DOR (﻿AFC < 4, FSH > 10 IU/ml, AMH < 1 ng/ml	101	75 mg/d	> 3 months	Long GnRH agonist	﻿Spontaneous pregnancies
Hyman et al. (63) *2013*	Prospective self-controlled	﻿At least one previous IVF cycle with ≤ 4 oocytes retrieved despite high dose Gn (≥ 450IU/day)	43	75 mg/d	>3 months	NR	NR
Singh et al. (64) *2013*	Prospective self-controlled	﻿Poor ovarian response in the previous IVF cycle(s)	31	75 mg/d	4 months	NR	AMH, FSH and antral follicle count
Yilmaz et al. (65) *2013*	Prospective	AFC <5 or AMH <1.1 ng/ml and a previous poor ovarian response	41	75 mg/d	> 6 weeks	﻿GnRH antagonist	AMH, Inhibin B and antral follicle count
Jirge et al. (66) *2014*	Prospective self-controlled	Bologna criteria <40ys with 1 previously failed IVF cycle	31	75 mg/d	> 2 months	﻿GnRH antagonist	﻿Dose and duration of gonadotropin therapy, oocyte yield, embryo number and quality, pregnancy and live birth rate.
Xu et al. (67) *2014*	Retrospective	Bologna criteria	386	75 mg/d	90 d	﻿GnRH antagonist	﻿Ongoing pregnancy rate and implantation rate
Zangmo et al. (68) *2014*	Prospective self-controlled	﻿<42 years, with <5 oocytes retrieved in previous IVF cycles, D2 FSH 10–20 mIU/ml	50	75 mg/d	4 months	NR	Oocyte and embryo number and quality
Tsui et al. (69) *2015*	Prospective self-controlled	Bologna criteria	10	90 mg/d	﻿12.2 weeks	﻿GnRH antagonist	﻿Total doses of rFSH, days of stimulation, oocytes retrieved, fertilized oocytes, Day 3 embryos, and transferred embryos
Vlahos et al. (70) *2015*	Prospective	At least 2 of the following: >40 years, D2 FSH >9.5 mIU/ml, AMH< 2 ng/ml, at least one previous cycle of COS with < 3 oocytes retrieved, at least one cancelled attempt owing to POR and E2 < 500 pg/ml on the day of trigger	161	75 mg/d	> 3 months	﻿GnRH antagonist	Live birth rate
Hu et al. (71) *2017*	Prospective	<40 years, subfertility >1 year, and DOR (two or more items such as FSH 10-25 IU/L, E2 >80 pg/ml, AMH <0.5-1.1 ng/ml and AFC ≤5 on cycle D2-3	106	75 mg/d	8 weeks	﻿GnRH antagonist	NR
Chern et al. (72) *2018*	Retrospective	Bologna criteria or 2 episodes of a previous POR after maximal stimulation alone	151	90 mg/d	3 months	﻿GnRH antagonist	Number of oocytes retrieved and clinical pregnancy rate
Al-Turki et al. (73) *2018*	Prospective	Bologna criteria, 25-40y with previously failed IVF cycle	62	50 mg/d	3 months	﻿GnRH antagonist	﻿Number of oocytes retrieved, fertilization rate, number of embryos and pregnancy rate
Wong et al. (74) *2018*	Prospective	POI	31	75 mg/d	12 months	NA	AMH
Chen et al. (75) *2019*	Retrospective	﻿POSEIDON group 4	297	90 mg/d	3 months	﻿GnRH antagonist	Number of oocytes retrieved and MII
Ozcil (76) *2020*	Retrospective	6 POI and 28 POR according to the Bologna criteria	34	50 mg/d	5 months	NA	Spontaneous clinical pregnancy rate

AFC, antral follicle count; AMH, antimullerian hormone; CI, confidence interval; COS, controlled ovarian stimulation; d, day(s); E2, estradiol; FSH, follicle stimulating hormone; Gn, gonadotropin; GnRH, gonadotropin releasing hormone; HCG, human chorionic gonadotropin; IVF, in vitro fertilization; MII, mature oocytes; NR, not reported; NA, not applicable; POI, premature ovarian insufficiency; POR, poor ovarian responders; y, years.

### Dehydroepiandrosterone

A case series of five patients with unexplained infertility and previous poor response to ovarian stimulation was the first study to analyze the effect of DHEA pretreatment on ovarian response (51). In this study, 80 mg/day of oral micronized DHEA was given for 2 months, after which ovarian stimulation was started with recombinant follicle stimulating hormone (rFSH) for intrauterine insemination. The authors concluded that oral DHEA supplementation might improve ovarian response and reduce gonadotrophin consumption. Five years later, a case report of a 43-years old patient seeking embryo accumulation for preimplantation genetic screening draw the scientific community’s attention to the role of androgens in ovarian response to stimulation (52). After her first stimulation cycle, the patient started self-administering 75 mg/day of oral micronized DHEA and initiated acupuncture treatment. In total, the patient performed 9 stimulation cycles with different stimulation protocols, and a significant increase in ovarian response was reported after four months of DHEA pretreatment. Since then, multiple observational and randomized controlled trials have followed, with varying DOR and poor ovarian reserve (POR) definitions, with DHEA doses ranging from 50 to 90 mg/day and a treatment duration ranging from 1 to 12 months, both before and during controlled ovarian stimulation (**Tables 1** and **2**). Importantly, no pharmacological studies have been performed to determine the optimal dose, duration or timing of DHEA supplementation in DOR patients.

Another key limitation regarding many studies on DHEA pre-treatment is the frequent use of patients as their own controls, comparing ovarian response after DHEA supplementation with a previous cycle. This study design does not take into account the importance of biological variability in the response to ovarian stimulation and the natural process of the regression to the mean, precluding definitive conclusions regarding the true effect of such treatment (77).

Also noteworthy is the fact that oral DHEA formulations are dietary supplements and therefore are not regulated by the US Food and Drug Administration (FDA) nor by the European Medicines Agency (EMA) and are exempt from pharmaceutical quality standards. Consequently, the true standardization of the formulations used cannot be guaranteed (78).

### Testosterone

Numerous observational and randomized controlled trials have also been published on the use of testosterone pre-treatment on POR and DOR patients (**Tables 1** and **2**). Most studies report the use of transdermal testosterone, both in gel and patches, with doses of treatment based on Vendola’s studies on primates (14, 15). In these studies, an effect on follicular development was reported with transdermal testosterone 20 µg/Kg/day, obtained with a 12.5mg/day gel application or a 2.5mg/day patch. Importantly, however, pharmacokinetics studies performed in postmenopausal women revealed that the administration of 4.4-5 mg testosterone gel or cream raised free testosterone levels within the reference range for reproductive-aged women whereas higher doses increased testosterone levels above the physiological range (79, 80). These findings question the potential clinical benefit (or harm) of using the high doses that have been reported so far.

The issue of the duration of treatment has also been another point of conflict in the published studies, ranging from 5 days, based on Vendola’s studies (14, 15), to 21-28 days, based on a RCT that reported that testosterone effects at the follicular level occurred after at least three weeks of testosterone pre-treatment (32). This should come as no surprise, if we consider that the progression from a primordial follicle to a periovulatory follicle takes approximately 3 months (81).

### Too Much Is Not Enough

**Figure 2:**
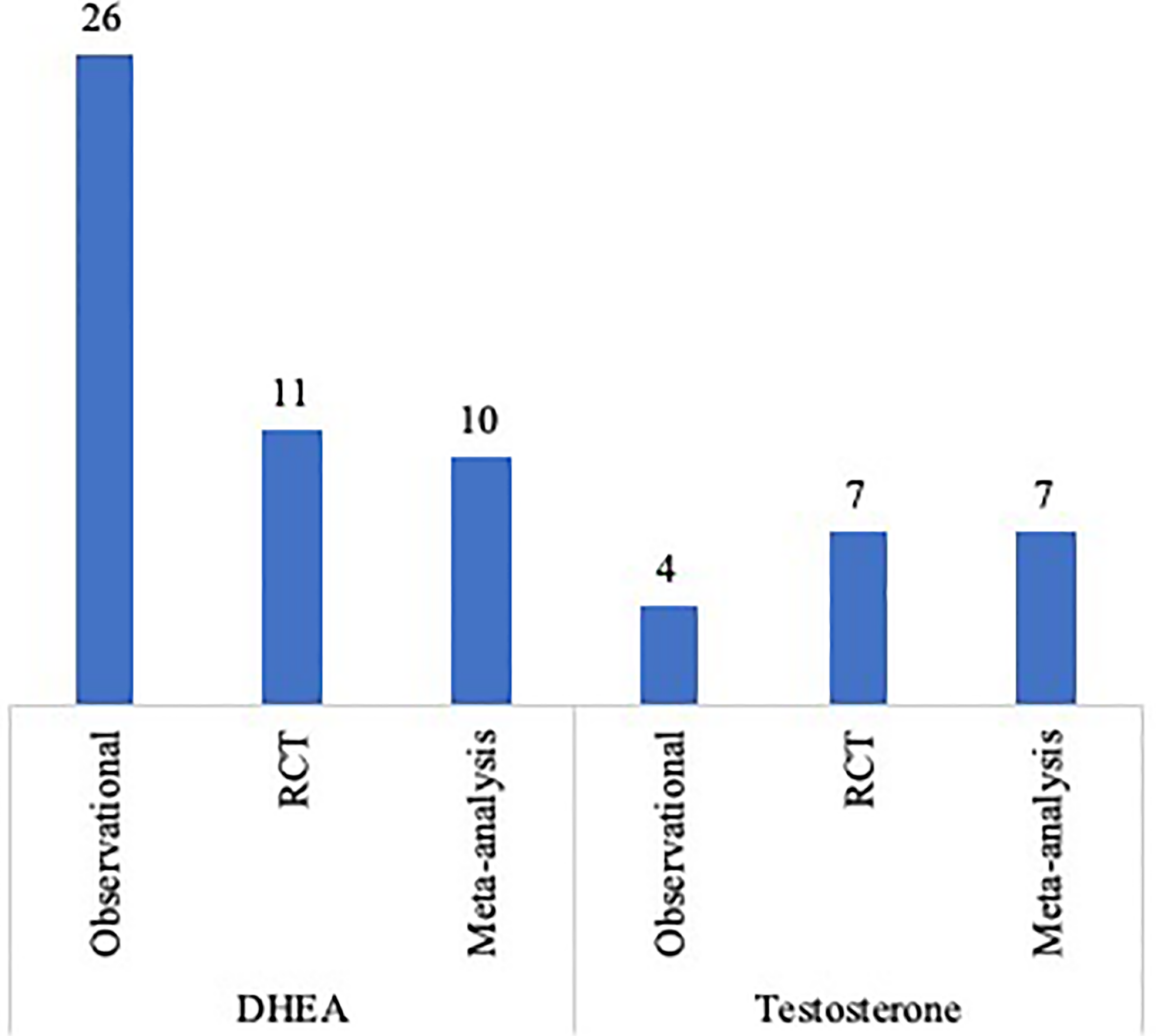
Published original studies and meta-analysis on the use of DHEA or testosterone supplementation in POR and DOR patients.

The vast bulk of published original studies and meta-analysis on the use of androgens pre-treatment in DOR and POR patients is depicted in **Figure 2**. One of the limitations in analyzing the effect of these adjuvant strategies in DOR/POR patients is the definition of diminished and poor response itself. In this context, the Poseidon Group introduced the concept of ‘low prognosis patients’ and highlighted the need for tailored evidence-based clinical algorithms for each of the four proposed risk groups (82, 83). Standardizing the inclusion criteria of future studies based on these risk groups might be a further step in minimizing study heterogeneity.

Despite the above-mentioned methodological limitations and the heterogeneity among the inclusion criteria and treatment protocols, original studies continue to be published in an attempt to optimize the clinical management of such a challenging population. With the same goal, a disproportionate number of meta-analysis has been published, especially when considering the number of original studies. **Table 3** describes the meta-analysis published on the use of DHEA and testosterone supplementation in IVF and the study design of the included trials. If we consider the low level of evidence of some of the included study designs, the lack of evidence-based protocols for both DHEA and testosterone supplementation, the heterogeneity in the definition of POR and DOR and the diversity in the IVF protocols used in the different trials, the clinical impact of the conclusions drawn from these meta-analysis might be called into question. In this regard, an individual patient data approach could be of use in increasing the strength of the available evidence.

**Table 3:** Published meta-analysis on the use of DHEA and Testosterone in IVF.

Author	Year	Number of studies	Population	Study design
**DHEA**				
Narckwichean et al. (84)	2013	3	DOR/POR	1 RCT, 2 Retrospective
Li et al. (85)	2015	8	DOR/POR	2 RCT, 2 Prospective, 4 Retrospective
Qin et al. (86)	2016	9	DOR/POR	4 RCT, 2 Prospective, 3 Retrospective
Liu et al. (87)	2017	6	NOR/DOR/POR	6 RCT
Schwarze et al. (88)	2018	5	DOR/POR	2 RCT, 1 Prospective, 2 Retrospective
Xu et al. (89)	2019	9	NOR/DOR/POR	9 RCT
**Testosterone**				
González-Comadran et al. (90)	2012	3	DOR/POR	3 RCT
Luo et al. (91)	2014	3	DOR/POR	3 RCT
Noventa et al. (92)	2019	7	DOR/POR	7 RCT
**Testosterone and DHEA**				
Sunkara et al. (93)	2011	5	DOR/POR	4 RCT, 1 Retrospective
Bosdou et al. (94)	2012	3	DOR/POR	3 RCT
Nagels et al. (95)	2015	17	NOR/DOR/POR/POI	17 RCT
Zhang et al. (96)	2019	4	POR	4 RCT

DHEA, dehydroepiandrosterone; DOR, diminished ovarian reserve; NOR, normoresponders; POI, premature ovarian insufficiency; POR, poor ovarian responders; RCT, randomized controlled trials.

However, to break this vicious cycle, we are left with the need to write the story of androgens supplementation in patients with DOR/POR from the beginning. In order to do so, evidence from pharmacokinetics studies (79) as well as from the timespan of human folliculogenesis (97) must be taken into account in what concerns the optimal dose and duration of treatment. In this respect, the currently ongoing multicenter ﻿double-blind placebo-controlled randomized controlled trial T-TRANSPORT ﻿(NCT02418572, available at http://clinicaltrials.gov/ct2/show/NCT02418572) might shed some light on this subject. With an intervention group undergoing 5.5 mg daily transdermal testosterone for two months prior to an IVF cycle and powered with clinical pregnancy rate as the primary outcome measure, this trial is expected to clarify the role of androgens in IVF.

## Conclusion

Despite the vast amount of available literature on the use of DHEA and testosterone in POR patients, the bulk of evidence is still limited to draw definite conclusions. More than reviewing the available data and publishing new studies based on the same pitfalls, we urge to restart this chapter with well-designed clinical trials.

## Author Contributions

AN designed the study, performed the literature review, contributed to the interpretation of the findings, wrote the manuscript and critically revised it. PM-B contributed to the interpretation of the findings and critically revised the manuscript. NP designed the study, supervised the writing of the manuscript, contributed to the interpretation of the findings and critically revised the manuscript. All authors contributed to the article and approved the submitted version.

## Conflict of Interest

NP is the principal investigator of the T-TRANSPORT trial.

The remaining authors declare that the research was conducted in the absence of any commercial or financial relationships that could be construed as a potential conflict of interest.
